# Stable sol–gel hydroxyapatite coating on zirconia dental implant for improved osseointegration

**DOI:** 10.1007/s10856-021-06550-6

**Published:** 2021-06-30

**Authors:** Jinyoung Kim, In-Gu Kang, Kwang-Hee Cheon, Sungmi Lee, Suhyung Park, Hyoun-Ee Kim, Cheol-Min Han

**Affiliations:** 1 Department of Materials Science and Engineering, Seoul National University, Seoul, 08826 Republic of Korea; 2 Biomedical Implant Convergence Research Center, Advanced Institutes of Convergence Technology, Suwon, 16229 Republic of Korea; 3 Department of Carbon and Nano Materials Engineering, Jeonju University, Jeonju, 55069 Republic of Korea

## Abstract

Aside from being known for its excellent mechanical properties and aesthetic effect, zirconia has recently attracted attention as a new dental implant material. Many studies have focused on hydroxyapatite (HA) coating for obtaining improved biocompatibility, however the coating stability was reduced by a byproduct produced during the high-temperature sintering process. In this study, to overcome this problem, we simply coated the zirconia surface with a sol–gel-derived hydroxyapatite (HA) layer and then sintered it at a varied temperature (<1000 °C). The surface showed a nanoporous structure, and there was no crystalline phase other than HA and zirconia when the sintering temperature was 800 °C. The adhesion strength of the HA layer (>40 MPa) was also appropriate as a dental implant application. In addition, in vitro cell experiments using a preosteoblast cell line revealed that the HA-coated zirconia surface acts as a preferable surface for cell attachment and proliferation than bare zirconia surface. In vivo animal experiments also demonstrated that the osteoconductivity of zirconia were dramatically enhanced by HA coating, which was comparable to that of Ti implant. These results suggest that the sol–gel-based HA-coated zirconia has a great potential for use as a dental implant material.

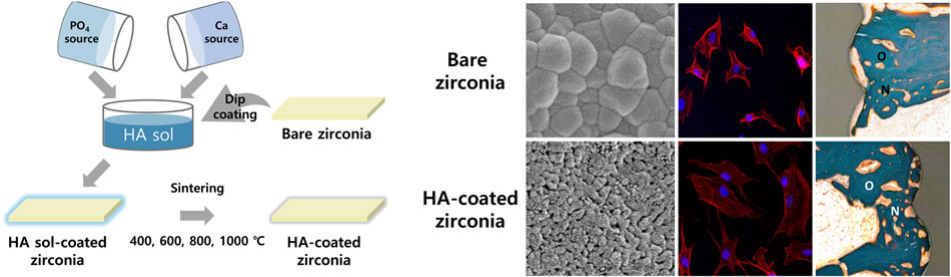

## Introduction

The past decades have seen constant demands for dental and bone-related implants according to the aging society [1, 2]. Titanium (Ti) and its alloys are the most widely used implant materials, which have excellent mechanical strength and biocompatibility [3, 4]. However, various problems have been reported when Ti is used as a tooth implant, including nonspecific immune reactions, autoimmune problems, and corrosion caused by body fluids, such as saliva [5]. Moreover, aesthetic problems caused by gray color of the Ti surfaces, which is very different to that of tooth, is also one of the most critical issues related to the Ti implant [6, 7].

Zirconia is bioinert ceramics which does not react with living tissues and causes immune reactions. Particularly, an yttria-stabilized zirconia demonstrates excellent fracture resistance and flexural strength. Due to its superior mechanical properties, zirconia has been used as ball head for total hip replacements [8–10]. In addition, it possesses an excellent aesthetic effect with almost the same color as the teeth [11, 12]. Thus, several attempts have recently been made to fabricate a dental implant using zirconia. However, poor osseointegration is one of the most serious drawbacks of zirconia as a biomaterial. Accordingly, many studies to overcome these problems through surface modification have been actively conducted in recent years [13–15].

The surface properties of zirconia are generally modified by coating it with osseointegrated materials, such as hydroxyapatite (HA). HA has been widely used for dental and orthopedic applications because of the similarity of their chemical compositions to the mineral phase of a bone. The effect of HA coating on bone regeneration have been proven by numerous in vitro and in vivo studies [16, 17]. One simple method to coat HA on zirconia implant surface is immersing the zirconia in Simulated Body Fluid (SBF). The HA layer precipitated in SBF demonstrated greatly increasing the cell characteristics, but the coating layer was not very stable due to the poor adhesion of the coating layer [18, 19]. Physical vapor deposition (PVD) is another way to coat HA on the zirconia surface. Since as-deposited HA layer is not fully crystallized, PVD methods generally require heat treatment at a high temperature above 1200 °C. HA and zirconia, however react above this temperature and produce a byproduct, namely calcium zirconate [20]. Calcium zirconate hinders the formation of a uniform HA coating layer because it was generated by consuming Ca in HA phase [21]. Kim et al. coated fluoric HA as a buffer layer to prevent this unwanted reaction [22]. However, coating of multiple layers might weaken the adhesion stability; thus, a method to coat a pure HA layer on zirconia without coating any intermediate layers must be developed.

Herein, a thin and stable HA layer was formed on a zirconia substrate using the sol–gel technique to improve its osseointegration. A sol–gel derived HA was coated on a zirconia substrate through the dip-coating method and then sintered with various sintering temperatures which is lower than that used in other studies (400–1000 °C). The microstructure and phases of the coating layer were examined by scanning electron microscopy (SEM) and X-ray diffraction (XRD), respectively. The bioactivity of the HA-coated zirconia was evaluated by an in vitro test using preosteoblast cells. Furthermore, an in vivo test using a rabbit femoral defect model was performed to assess the osteoconductivity of the HA-coated zirconia dental implant.

## Materials and methods

### Preparation of the zirconia substrates

A plate-type zirconia substrate was used to optimize the coating conditions and evaluate the in vitro cellular behaviors. A zirconia block was produced by the following process: first, zirconia stabilized by 3 mol% of yttria powder (3Y-TZP, Tosoh Corporation, Japan) was compressed at 20 MPa, followed by the application of cold isostatic press at a pressure of 200–300 MPa and pre-sintering at 1100 °C; next, the block was cut into a plate shape with dimensions of 10 × 10 × 1 mm^3^; and the zirconia plate was then sintered at 1500 °C for 2 h at a heating rate of 5 °C/min. A screw-type zirconia substrate was also prepared by machining the zirconia block to screw shape with 2-mm diameter and 10-mm length to evaluate the osteoconductivity under the in vivo condition.

### HA sol synthesis

First, 0.015 mol (2.494 g) of triethylphosphite (TEP, Sigma-Aldrich, USA) was added to a mixed solution of 23.92 ml ethanol (Daejung, Korea) and 1.08 ml deionized water. Second, 0.0251 mol (4.1102 g) of calcium nitrate tetrahydrate (Ca(NO_3_)_2_ · 4H_2_O, Sigma-Aldrich, USA) was dissolved in 25 ml ethanol. These solutions were mixed together at room temperature for 3 days and aged at 37 °C for 1 day with continuous stirring to make the HA sol. The theoretical concentration of the final HA sol is 0.05 M when it is assumed that all reactants have completely reacted. The synthesis of the HA sol was confirmed by observing the crystallized nanoparticles through transmission electron microscope (TEM; Tecnai F20, FEI, USA) (Fig. S1).

### HA coating on zirconia

HA was coated on the zirconia plate and screw using the dip-coating technique. The dip-coating rate was 0.25 mm/s and performed thrice. The sol was dried for 5 min in the oven at 37 °C between each dipping step. The HA-coated zirconia plate was oven-dried at 70 °C overnight and sintered at 400 °C, 600 °C, 800 °C, and 1000 °C for 1 h at a heating rate of 5 °C/min after the coating was completed. The specimens generated under these conditions were designated as HA400, HA600, HA800, and HA1000, respectively. For the zirconia screw for using in vivo study, excessive sol was trapped between the screw threads, which might cause an unwanted thick coating layer; thus, spinning was performed at 150 rpm for 15 s using a spin coater after every dip-coating. Subsequently, the HA-coated zirconia screw was heat-treated at 800 °C for 1 h at a heating rate of 5 °C/min.

### Characterization of the HA-coated zirconia

The surface microstructure of the HA-coated zirconia was analyzed using a field emission scanning electron microscope (FE-SEM; Supra 55VP, Carl Zeiss, Germany). Prior to the FE-SEM observation, a thin platinum layer was deposited (EM ACE200, Leica, Austria) to prevent electron charging. The focused ion beam (FIB, Aurica, Zeiss, Germany) technique was used to analyze the coating layer thickness. The crystal phase of the HA-coated zirconia was investigated by XRD (D8 advance, Bruker Miller co, Germany). Wide 2θ angle range of 20°–60° and narrow 2θ angle range of 31°–37° was obtained at a scan rate of 1 °/min because of its small intensity. The binding energy of composing elements of HA-coated zirconia was measured by X-ray photoelectron spectroscope (XPS; AXIS-HSi, KRATOS, Japan).

The mechanical stability of the coating layer was measured using the pull-out method. Epoxy precoated aluminum studs were placed on the coating surface and cured at 150 °C for 30 min. The stud was pulled from the attached surface at a rate of 1 mm/min using a universal testing machine (RB302 single-column type, R&B, Korea). In order to evaluate the stability of the coating layer in the screw-shaped specimen, the HA-coated screw-shaped specimen was removed after implantation by applying a torque of 0.4 N·m to the synthetic bone, and the surface of the implanted part and the unplaced part was observed through SEM.

### In vitro cell tests

The initial cell attachment and proliferation were evaluated using preosteoblast cell lines (MC3T3-E1, ATCC, CRL-2593, USA). All specimens were sterilized with 70% ethanol solution and UV radiation prior to cell seeding. The pre-incubated preosteoblast cell lines were seeded on each sample at densities of 3 × 10^4^ and 2 × 10^4^ cells/ml for the cell attachment and proliferation assays, respectively. The cells were then incubated in a humidified incubator at 37 °C with 5% CO_2_. The alpha-minimum essential medium (α-MEM, Welgene Co, Korea) containing 10% fetal bovine serum (Gibco, USA) and 1% penicillin–streptomycin (Pen strep, Gibco, USA) was used as the culturing medium for all tests.

The initial cell adhesion was observed using a confocal laser scanning microscope (SP8 X, Leica, Germany) 24 h after cell seeding. After fixing the attached cells with 4% paraformaldehyde solution, the cytoplasm and nuclei of the cells were stained with fluorescent phalloidin (Alexa Fluor^®^ 555 phalloidin, Invitrogen, USA), and 4′,6-diamidino-2-phenylindole (DAPI, Invitrogen, USA) and then observed using lasers with wavelengths of 540 and 350 nm, respectively.

The cell proliferation was assessed using the metoxyphenyl tetrazolium salt (MTS) method. A 10% reagent (CellTiter 96 Aqueous One Solution, Promega, USA) in culture medium was added to the cells on the specimens and incubated at 37 °C for 2 h after 3 and 5 days of culturing. The supernatant absorbance was measured by a microplate reader (EZ read 400, Biochrom, UK) at 490 nm.

### In vivo animal test

An animal test was conducted using a rabbit femoral defect model. This animal test was approved by Genoss’s Institutional Animal Care and Use Committee. Twelve-week-old male New Zealand white rabbits with an average weight of 2.5–3 kg were anesthetized with 0.1 ml of 2% Xylazine HCl (Rompun, Bayer Korea, Korea) and 0.2 ml Tiletamine HCl (Zoletil, Virbac lab, France), and Lidocaine (Yuhan Corporation, Korea). A cylindrical defect with a radius of 0.9 mm was created on each leg. Zirconia and HA-coated zirconia screws and Ti screw, which are positive control, were then implanted to these defects by applying an insertion torque of 20 Ncm. A postoperative antibiotic, gentamicin (Dongkwang Pharmaceutical Co, Korea), was intramuscularly administered at 0.1 mg/kg for 3 days after surgery.

The rabbits were sacrificed four weeks after the surgery; further, the specimens were extracted from the surrounding tissues. For a histological observation, all the extracted specimens were immediately fixed with 10% formalin then embedded in resin (Technovit 7200 VLC, Germany). The resin blocks containing the specimen and the tissues were sliced to a thickness of 40 μm. The slides were then stained using Goldner’s trichrome method and observed using an optical microscope (Olympus BX51, Olympus, Japan). The osteoconductivity was evaluated in terms of the bone-to-implant contact (BIC) and new bone area ratios. The BIC ratio was calculated by dividing the length of the screw surface that bonded with the bone by the whole length of the screw surface. In addition, the new bone area ratio was defined as the area of the newly generated bone relative to the area inside the threads at the top part of the screw.

### Statistical analysis

Statistical Package for the Social Sciences (SPSS 23, SPSS Inc., USA) was used to perform the statistical analysis. All data were expressed as mean ± standard deviation. The normality of the variables was verified using the Shapiro–Wilk test. The statistical analysis was performed by a one-way analysis of variance with Tukey post-hoc comparison. A *p* value below 0.05 was considered significant in all cases.

## Results

### Surface morphology of the HA layer

**Fig. 1:**
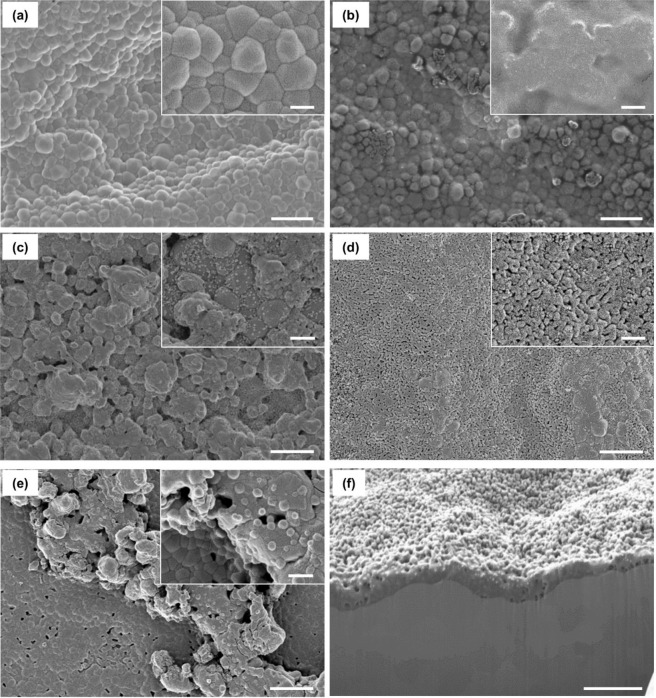
SEM images of the HA-coated zirconia substrates with various sintering temperatures. **a** Bare zirconia, **b** HA400, **c** HA600, **d** HA800, **e** HA1000, and **f** cross-sectional image of HA800. Scale bars: **a**–**e** 2 μm and 500 nm for large and inset images, respectively. **f** 1 μm

Figure 1 shows the surfaces of the bare and HA-coated zirconia with various sintering temperatures observed by SEM. Figure 1(a) depicts that the bare zirconia had a dense surface, and the average grain size was 500 nm. For HA400, zirconia grains were observed through the translucent layer, which did not have any grain structure (Fig. 1b). Meanwhile, small granules of HA were observed for HA600 (Fig. 1(c)). Most of the granules had a dense structure, while a few granules showed very small pores. The surface became flat when the sintering temperature increased to 800 °C (Fig. 1d). Interestingly, the inset of Fig. 1d demonstrates a nanoporous structure. The average pore size of the HA800 was ~50 nm. Figure 1e shows HA1000. Most of the zirconia surface was covered with the flat HA layer, but the coating uniformity decreased because of some HA agglomerates. The nanoporous structure also disappeared. Figure 1f shows the cross-sectional image of the HA800 sectioned by the FIB technique. The thickness of the HA layer was very uniform through the surface, and its average value was ~400 nm, which was much thinner than that in the previous studies [7, 23].

### Characteristics of the HA-coated zirconia

**Fig. 2:**
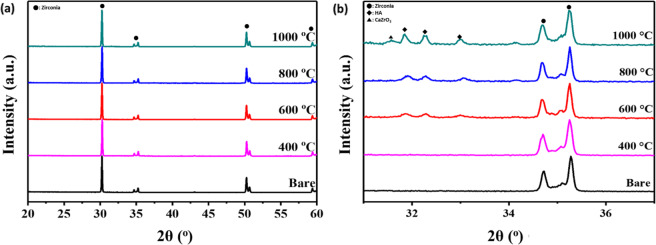
XRD patterns of the HA-coated zirconia substrates with various sintering temperatures. Range of 2θ are **a** 20°–60° and **b** 31°–37°

The crystalline phases of the HA layers with different sintering temperatures were investigated by XRD (Fig. 2). The wide range (20°–60°) XRD spectra, shown in Fig. 2a only demonstrated characteristic peaks of zirconia substrate (ICDD data file no. 01-072-2743), since the coating layer was very thin. In order to confirm the other component, the narrower range (31°–37°) of XRD pattern was further investigated as shown in Fig. 2b. In the narrow range spectra, two main peaks on the bare zirconia substrate were detected at 34.7° and 35.3°, which corresponded to the (110) and (002) planes of tetragonal zirconia, respectively. A broad and small peak corresponding to the (200) plane of cubic zirconia was detected at around 35°. The XRD pattern of HA400 also demonstrated the characteristic peaks of zirconia but did not show any calcium phosphate peak. The HA peaks (ICDD data file no. 04-014-8416) began to be detected at 31.9°, 32.2°, and 33° corresponding to (211), (112) and (300) planes, respectively, when the sintering temperature was above 600 °C. The intensity of the HA peaks also increased when the sintering temperature increased. However, for HA1000, aside from the sharper and higher HA peaks, calcium zirconate phase (ICDD data file no. 04-001-9308) was also detected at 31.7°.

### Mechanical stability of the HA coating layer

**Fig. 3:**
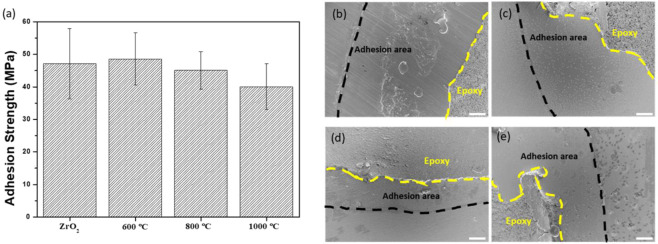
Adhesion strength measured through peel-off test and SEM images after testing. Adhesion strength of the HA-coated zirconia substrates with various sintering temperatures **a**. SEM images of the failure surfaces of the bare and HA-coated zirconia after the adhesion test: **b** bare zirconia, **c** HA600, **d** HA800, and **e** HA1000. The black and yellow dotted lines indicate the areas where the stud adheres to and epoxy remains, respectively. Scale bars: 500 nm

Figure 3a shows the adhesion strength of HA layers examined by peel-off test, which is widely used to evaluate the adhesion strength of HA coating layer [22–24]. Bare zirconia also measured under the same conditions to determine the bonding strength of stud and epoxy. HA400 was excluded because it was not suitable for coating purpose, as described in the earlier sections. All sample groups demonstrated the adhesion strength higher than 40 MPa without a statistical difference. After the peel-off test, the failed region was investigated using SEM observation (Fig. 3b–e). For all specimen surfaces, epoxy resin remained on the most of surface, and delamination of HA layer was not observed even epoxy resin was removed.

### In vitro biocompatibility of the HA-coated zirconia

**Fig. 4:**
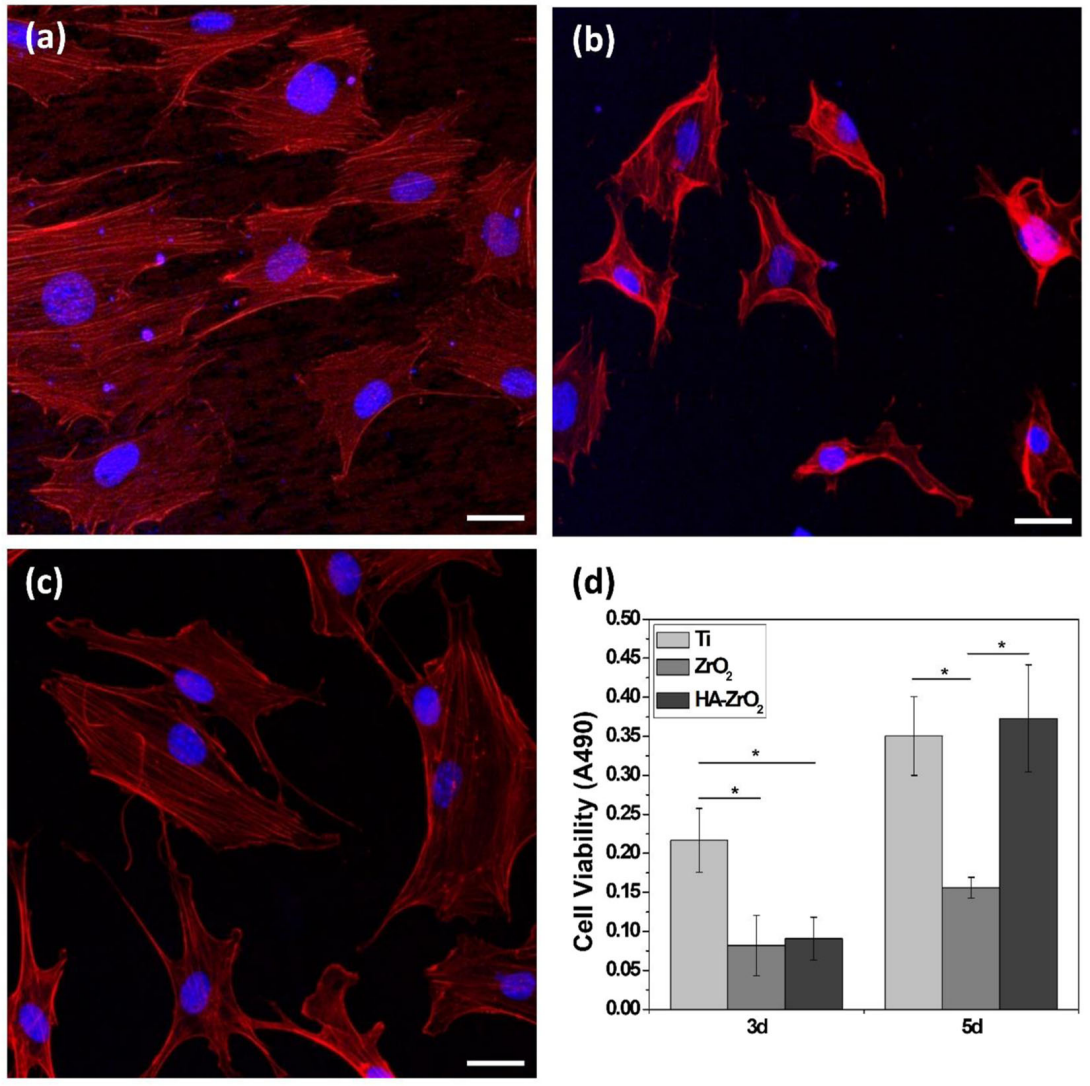
In vitro cell attachment and cell proliferation. Confocal laser scanning microscope image of cells attached on the **a** Ti, **b** bare, and **c** HA-coated zirconia surfaces. **d** Degree of proliferation of the MC3T3-E1 cells on the Ti, bare, and HA-coated zirconia substrates after 3 and 5 days of seeding. Scale bars: confocal image (30 μm)

The effects of HA coating on the biological properties of zirconia were evaluated in terms of the initial cell attachment and proliferation. For comparison in the in vitro test, cell behavior was also evaluated on the Ti substrate, which has been most widely used in the dental implant field. The CLSM images depicted the cell morphology on the Ti, bare, and HA-coated zirconia (HA800) (Fig. 4a–c, respectively). Predictably, the cells were well attached and spread along the polishing line on the Ti substrate. A smaller number of cells was observed for the bare zirconia and the spreading of cells was also suppressed. Meanwhile, the number of cells for HA800 increased, and each cell was widely spread.

Figure 4d shows the degree of preosteoblast proliferation on each substrate evaluated by MTS. After 3 days of culturing, the cell proliferation on the Ti substrate was observed to be much faster than that on the other substrate. No significant difference was found between the bare zirconia and HA800. The degree of proliferation of the preosteoblast cell on HA800 dramatically increased after 5 days of culturing, which was similar to that of the Ti substrate.

### In vivo osteoconductive properties of the HA-coated zirconia

**Fig. 5:**
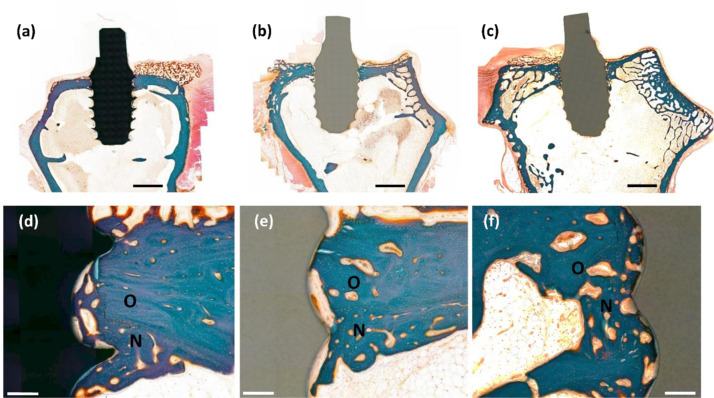
Representative histological cross-sectional image of the stained slices after 4 weeks of implantation. **a**, **d** Ti, **b**, **e** bare, and **c**, **f** HA-coated zirconia. O and N indicate the old and new bone areas, respectively. Scale bars: **a–c** low-magnification image (2 mm) and (**d–f** high magnification images (200 μm).

Animal experiments were performed to confirm the effect of the HA coating on the in vivo osteoconductivity of the zirconia-based implant and Ti implant for comparison purposes. Figure 5 shows the typical histological images of trichrome stained slices after 6 weeks of implantation. The blue, brown, and black regions in the histological images indicate the bone, zirconia implant, and Ti implant, respectively. In the low-magnification images, no signs of inflammation caused by implantation were observed for all the sample groups (Fig. 5a–c). Meanwhile, the differences between each sample group were clearly observed in the enlarged images. First, only a few contacted areas between the bone and the implant were observed for the bare zirconia implant, and a little amount of bone filled the space inside the screw thread. After the HA coating, a larger amount of bone tissue contacted with the implant surface and filled the space inside the thread. The in vivo osteoconductivity of the HA-coated zirconia was also compared with that of a commercially available Ti implant. The Ti implant provided a similar level of bone contact and filling to the HA-coated zirconia.

**Fig. 6:**
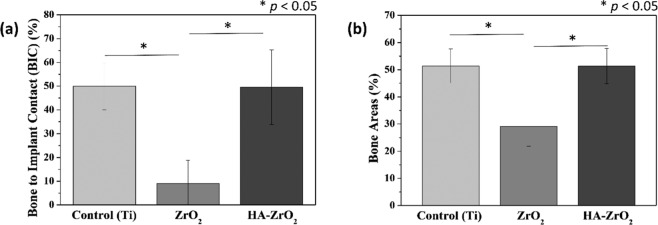
Quantitative analyses of in vivo experiments. **a** Bone-to-implant contact and **b** new bone area ratios of Ti, bare-, and HA-coated zirconia (*p < 0.05)

The BIC and BA ratios were investigated (Fig. 6) to quantify the in vivo osteoconductivity of the bare zirconia, HA-coated zirconia, and Ti implants. Figure 6a depicts that the HA-coated zirconia has a similar value of approximately 50% compared with Ti, while the bare zirconia has a BIC ratio value <10%. This result is almost the same as the image of the histological analysis identified earlier. Figure 6b illustrates a similar pattern when the bone areas were examined. The HA-coated zirconia was ~50%, which was about the same as 50% of Ti and significantly larger than zirconia, which is ~30%.

## Discussion

Zirconia is mechanically and chemically stable ceramic biomaterials. Zirconia also has tooth like color so that it is used as an abutment, but its bioinertness has limited the application as a fixture. HA coating is one of the simplest ways to enhance the osteoconductivity of zirconia for dental implants. Mechanical stability of coating layer is also important for implant materials because initial micromovement of implant is key factor for the implant success [25, 26]. In this study, the structure and crystal phase of the sol–gel based HA coating layer were controlled by adjusting the sintering temperature. The optimized HA coating demonstrated superior coating stability as well as osseointegration without showing byproduct phases, which is one of major problems of HA-coated zirconia system.

The morphological change in HA coating layer with different sintering temperature could be explained by the mobility of ions. As the sintering temperature increases, the mobility of ions also increases. The higher mobility led to a faster reaction between the HA particles. For HA600, adjacent HA particles began to merge together and make granular structures on the zirconia surface. The porous structure of HA800 might be caused by the atomic diffusivity being not high enough to make the dense surface. Likewise, most of the HA1000 specimen surface was covered with the flat and dense HA layer having a large grain size owing to its high atomic diffusivity. In addition, the pores were found in the HA layer inside and in the HA layer surface, suggesting that they would have an interconnected structure. The micro-roughness of the implant surface is known to be highly correlated with the bone integration of the materials [27]. Based on these results, it is considered that the HA800 group has most osteoconductive surface due to its micro-roughened surface.

Analysis of the crystallinity of HA coated zirconia revealed more information. The absence of any peaks in XRD observations means that the HA layer does not crystallize at sintering temperature conditions as low as 400 °C. Amorphous HA has a very high dissolution rate; hence, sintering at 400 °C would not be appropriate for the coating purpose to improve the osteointegration of dental implants [28]. Calcium zirconate observed in XRD analysis of specimens fabricated at a sintering temperature of 1000 °C is an undesirable reactant of HA and zirconia because it consumes calcium and reduces the Ca and P ratio in the calcium phosphate. During heat treatment, tricalcium phosphate is produced by the thermal decomposition of HA, as shown in Eq. (1). This usually occurs above 1300 °C. However, when zirconia is present, the reaction temperature could be lowered to 1000–1150 °C because the reaction occurs as Eq. (2), and calcium zirconate is formed as a byproduct [29–31]. This phenomenon occurs at high temperatures because it provides sufficient mobility and time for the decomposition of HA to TCP and the reaction of CaO, a byproduct of decomposition, and zirconia [32]. The effect of sintering temperature on the chemical composition of coating layer was further carried out through X-ray photoelectron spectroscopy (Fig. S2). The survey spectra showed that the characteristic peak of Zr is only observed in HA1000 (Fig. S2a, b). In addition, comparing the O 1 s spectra, the binding energy corresponding to metal oxide was observed only in HA1000 (Fig. S2c, d). Because the XPS method can normally observe a depth of 10 nm, these Zr and metallic oxide peaks would be obtained not from the substrate but from a byproduct, such as calcium zirconate. The calcium zirconate, which is an unwanted by-product that is finally produced, not only degrade the mechanical properties, but also lower the osseoconductivity of the coating layer [33, 34].

1 $${\mathrm{Ca}}_{10}\left( {{\mathrm{PO}}_4} \right)_6\left( {{\mathrm{OH}}} \right)_2 \to 3{\mathrm{Ca}}_3\left( {{\mathrm{PO}}_4} \right)_2 + {\mathrm{CaO}} + {\mathrm{H}}_2{\mathrm{O}}$$

2 $${\mathrm{Ca}}_{10}\left( {{\mathrm{PO}}_4} \right)_6\left( {{\mathrm{OH}}} \right)_2 + {\mathrm{ZrO}}_2 \to 3{\mathrm{Ca}}_3\left( {{\mathrm{PO}}_4} \right)_2 + {\mathrm{CaZrO}}_3 + {\mathrm{H}}_2{\mathrm{O}}$$

These results suggest that the HA coating layer should be heat-treated at a temperature between 400 °C and 1000 °C to obtain the crystalline HA phase without any by-products.

The mechanical stability of the coating layer is a very important factor for the success of the implantation. The coating layer is not effective anymore if it is peeled off during the surgery. In addition, delaminated debris could induce unwanted bone regeneration [35]. Weak bonding strength between coating and substrate also can cause micromovement of implant which might result in implant failure [25, 26]. From this point of view, the adhesion strength measured by the peel off test shows that the mechanical stability of the HA coating could be reasonable for dental applications. The measured strength must be lower than the actual adhesion strength between the HA layer and zirconia substrates because the separation of the HA layer is not seen when observing the area after measurement. In this way, several existing studies have measured the coating strength of various materials, which is similar to or lower than the value of 40 MPa in this study [36–38]. According to ISO standard (ISO 13779) and researches, adhesion strength higher than 15 MPa is considered acceptable [24]. In addition, it was confirmed that the bonding strength measured in a similar method in the previous papers on HA coated zirconia sintered at a high temperature of 1600 °C has a value of 11.68 MPa [39, 40]. This was thought to be a phenomenon caused by the decrease in mechanical properties due to the formation of TCP and calcium zirconate at high temperatures mentioned above, and this was proved through the observation of TCP in XRD analysis of the fracture surface. Therefore, HA coated zirconia obtained through sintering at a relatively low temperature had a bonding strength superior to existing studies as well as international standards, making it suitable for implant application. In addition, the stability of coating layer on the screw type specimen was also investigated. Because the use of stud is not applicable to the screw type specimen, the coating stability was confirmed by SEM after implantation the HA-coated zirconia on synthetic bone (Fig. S3). There was no noticeable crack on the coating layer both at ridge and groove parts of screw indicating that the coating layer on the screw is also mechanically stable. Collectively, 800 °C was chosen as the optimum sintering temperature for further biological test because HA800 showed the most ideal characteristics in terms of surface morphology, crystallinity, and coating stability.

The improved osteoconductivity of HA-coated zirconia was examined by in vitro cell test and in vivo animal study. The biocompatibility of Ti was also evaluated as a positive control because it is most widely used as dental implant [11]. The preosteoblast cells did not stretch well on the bare zirconia substrate. This is mainly due to the zirconia properties known as bioinert [41, 42]. On the other hand, HA800 sample showed good cell extension, which is similar to the cells attached on Ti (Fig. 4a, c). In addition, proliferation level of preosteoblast cells was also improved on HA800 compared with that on bare zirconia. Difference in proliferation level between HA800 and Ti groups was not statistically significant at day 5. The animal experiment revealed the improved osteoconductivity of HA-coated zirconia more directly. HA-coated zirconia and Ti showed almost the same behaviors in histological image, BIC and BA. BIC and BA of HA800 group were ~400 and 80% higher than bare zirconia group, respectively. Proven by cell and animal experiments, once the zirconia is coated with HA, the biocompatibility, especially osteoconductivity, is considerably increased compared with zirconia without HA coating. These findings suggest that the sol–gel-based HA-coated zirconia implant has a good potential as dental implant material.

## Conclusion

Herein, sol–gel derived HA was successfully coated on the zirconia surface using a simple dip-coating technique to improve its osseointegration. The surface morphology and the crystalline phase of the HA layer were varied with the sintering temperature. A nanoporous HA layer was uniformly formed on the zirconia substrate when the sintering temperature was 800 °C. The average thickness and pore size of the HA layer were ~400 nm and ~50 nm, respectively. In addition, there was no cracks on the coating layer, and it was not easily detached under 40 MPa of stress. The HA-coated zirconia exhibited a significantly enhanced osteoconductivity. The MC3T3-E1 preosteoblast cells more actively attached and proliferated on the HA-coated zirconia compared with the bare zirconia surface. The HA-coated zirconia dental implant sample demonstrated a remarkably improved in vivo osteoconductivity similar to that of the Ti implant. Collectively, these results suggest that a low-temperature sintered sol–gel-derived HA coating would increase the potential of zirconia in dental applications.

## Supplementary information

Supplementary Materials

## References

[1] Sykaras N, Iacopino AM, Marker VA, Triplett RG, Woody RD. Implant materials, designs, and surface topographies: their effect on osseointegration. A literature review. Int J Oral Maxillofacial Impl. 2000;15.675–90. 11055135

[2] Schwitalla A, Müller W-D (2013). PEEK dental implants: a review of the literature. J Oral Implant.

[3] Mohseni E, Zalnezhad E, Bushroa AR (2014). Comparative investigation on the adhesion of hydroxyapatite coating on Ti–6Al–4V implant: A review paper. Int J Adhesion Adhesives.

[4] Le Guehennec L, Soueidan A, Layrolle P, Amouriq Y (2007). Surface treatments of titanium dental implants for rapid osseointegration. Dental Mater.

[5] Ozkurt Z, Kazazoglu E (2011). Zirconia dental implants: a literature review. J Oral Implantol.

[6] Heydecke G, Kohal R, Gläser R. Optimal esthetics in single-tooth replacement with the Re-Implant system: a case report. Int J Prosthodont. 1999;12.184–9. 10371922

[7] Cho Y, Hong J, Ryoo H, Kim D, Park J, Han J (2015). Osteogenic responses to zirconia with hydroxyapatite coating by aerosol deposition. J Dental Res.

[8] Piconi C, Burger W, Richter H, Cittadini A, Maccauro G, Covacci V, et al. Y-TZP ceramics for artificial joint replacements. Biomaterials. 1998;19:1489–94. 10.1016/s0142-9612(98)00064-7. 10.1016/s0142-9612(98)00064-7 9794524

[9] Rahaman MN, Yao A, Bal BS, Garino JP, Ries MD (2007). Ceramics for prosthetic hip and knee joint replacement. J Am Ceramic Soc.

[10] Zhu Y, Liu K, Deng J, Ye J, Ai F, Ouyang H (2019). 3D printed zirconia ceramic hip joint with precise structure and broad-spectrum antibacterial properties. Int J Nanomed.

[11] Depprich R, Zipprich H, Ommerborn M, Naujoks C, Wiesmann H-P, Kiattavorncharoen S (2008). Osseointegration of zirconia implants compared with titanium: an in vivo study. Head Face Med.

[12] Gahlert M, Gudehus T, Eichhorn S, Steinhauser E, Kniha H, Erhardt W (2007). Biomechanical and histomorphometric comparison between zirconia implants with varying surface textures and a titanium implant in the maxilla of miniature pigs. Clin Oral Implants Res.

[13] Denry I, Kelly JR (2008). State of the art of zirconia for dental applications. Dental Mater.

[14] Siddiqui DA, Jacob JJ, Fidai AB, Rodrigues DC (2019). Biological characterization of surface-treated dental implant materials in contact with mammalian host and bacterial cells: titanium versus zirconia. RSC Adv.

[15] Liu YT, Lee TM, Lui TS (2013). Enhanced osteoblastic cell response on zirconia by bio-inspired surface modification. Colloids Surf B: Biointerf.

[16] Ong JL, Chan DCN (2000). Hydroxyapatite and their use as coatings in dental implants: a review. Criti Rev Biomed Eng.

[17] Kong YM, Kim DH, Kim HE, Heo SJ, Koak JY (2002). Hydroxyapatite-based composite for dental implants: an in vivo removal torque experiment. J Biomed Mater Res.

[18] Oyane A, Kakehata M, Sakamaki I, Pyatenko A, Yashiro H, Ito A (2016). Biomimetic apatite coating on yttria-stabilized tetragonal zirconia utilizing femtosecond laser surface processing. Surf Coatings Technol.

[19] Quan H, Park YK, Kim SK, Heo SJ, Koak JY, Han JS (2016). Surface characterization and human stem cell behaviors of zirconia implant disks biomimetic-treated in simulated body fluid. Int J Oral Maxillofac Implants.

[20] Hasegawa A, Kameyama T, Motoe A, Ueda M, Akashi K, Fukuda K (1992). Coating of hydroxyapatite on zirconia utilizing a radio-frequency thermal plasma process. J Ceramic Soc Jpn.

[21] Chiu C-Y, Hsu H-C, Tuan W-H (2007). Effect of zirconia addition on the microstructural evolution of porous hydroxyapatite. Ceramics Int.

[22] Kim H-W, Lee S-Y, Bae C-J, Noh Y-J, Kim H-E, Kim H-M (2003). Porous ZrO2 bone scaffold coated with hydroxyapatite with fluorapatite intermediate layer. Biomaterials.

[23] Sakthiabirami K, Vu VT, Kim JW, Kang JH, Jang KJ, Oh GJ (2018). Tailoring interfacial interaction through glass fusion in glass/zinc-hydroxyapatite composite coatings on glass-infiltrated zirconia. Ceramics Int.

[24] Zhang S, Wang YS, Zeng XT, Khor KA, Weng W, Sun DE (2008). Evaluation of adhesion strength and toughness of fluoridated hydroxyapatite coatings. Thin Solid Films.

[25] Szmukler‐Moncler S, Salama H, Reingewirtz Y, Dubruille J (1998). Timing of loading and effect of micromotion on bone–dental implant interface: review of experimental literature. J Biomed Mater Res.

[26] Wazen RM, Currey JA, Guo H, Brunski JB, Helms JA, Nanci A (2013). Micromotion-induced strain fields influence early stages of repair at bone-implant interfaces. Acta Biomaterialia.

[27] Wennerberg A, Albrektsson T (2009). Structural influence from calcium phosphate coatings and its possible effect on enhanced bone integration. Acta Odontologica Scandinavica.

[28] LeGeros RZ. Properties of osteoconductive biomaterials: calcium phosphates. Clin Orthopaedics Rel Res. 2002;395:81–98. 10.1097/00003086-200202000-00009. 10.1097/00003086-200202000-00009 11937868

[29] Evis Z (2007). Reactions in hydroxylapatite–zirconia composites. Ceramics Int.

[30] Kim HW, Kong YM, Koh YH, Kim HE, Kim HM, Ko JS (2003). Pressureless sintering and mechanical and biological properties of fluor‐hydroxyapatite composites with zirconia. J Am Ceramic Soc.

[31] Kim H-W, Noh Y-J, Koh Y-H, Kim H-E, Kim H-M (2002). Effect of CaF2 on densification and properties of hydroxyapatite–zirconia composites for biomedical applications. Biomaterials.

[32] Khor K, Fu L, Lim V, Cheang P (2000). The effects of ZrO2 on the phase compositions of plasma sprayed HA/YSZ composite coatings. Mater Sci Eng A.

[33] Ben Ayed F, Bouaziz J (2008). Sintering of tricalcium phosphate–fluorapatite composites with zirconia. J Eur Ceramic Soc.

[34] Klein C, Driessen A, De Groot K, Van Den Hooff A (1983). Biodegradation behavior of various calcium phosphate materials in bone tissue. J Biomed Mater Res.

[35] Revell P, Al-Saffar N, Kobayashi A (1997). Biological reaction to debris in relation to joint prostheses. Proc Inst Mech Eng Part H: J Eng Med.

[36] Zheng X, Huang M, Ding C (2000). Bond strength of plasma-sprayed hydroxyapatite/Ti composite coatings. Biomaterials.

[37] Liu D-M, Yang Q, Troczynski T (2002). Sol–gel hydroxyapatite coatings on stainless steel substrates. Biomaterials.

[38] Weng W, Baptista JL (1999). Preparation and characterization of hydroxyapatite coatings on Ti6Al4V alloy by a sol-gel method. J Am Ceramic Soc.

[39] Quan R, Zhang L, Xu J, Wang C, Wei X, Yang D (2015). The Bonding Strength of HA/ZrO2-Layered Biomaterials with Different Interfacial Patterns. Int J Appl Ceramic Technol.

[40] Wei X, Wang C, Li Y, Quan R, Lai Y (2016). Bonding strength of HA/ZrO2 biomaterials with different interlayers. Composite Interfaces.

[41] Matsumoto TJ, An SH, Ishimoto T, Nakano T, Matsumoto T, Imazato S (2011). Zirconia-hydroxyapatite composite material with micro porous structure. Dental Mater.

[42] Daisuke Y, Hideo S, Motoharu M, Seiji B (2008). Hydroxyapatite coating on zirconia using glass coating technique. J Ceramic Soc Jpn.

