# Supplementary material for: Stable sol–gel hydroxyapatite coating on zirconia dental implant for improved osseointegration
Source: J Mater Sci Mater Med. 2021 Jun 30;32(7):81. doi: 10.1007/s10856-021-06550-6 (PMC8245356; doi:10.1007/s10856-021-06550-6)
Supplement: Supplementary file 1 — Supplementary Materials [file 10856_2021_6550_MOESM1_ESM.docx]

**Stable sol–gel hydroxyapatite coating on zirconia dental implant for improved osseointegration**

*Jinyoung Kim^1^, In-Gu Kang^1^, Kwang-Hee Cheon^1^, Sungmi Lee^2^, Suhyung Park^1^, Hyoun-Ee Kim^1^, Cheol-Min Han^*,3^*

^1^Department of Materials Science and Engineering, Seoul National University, Seoul 08826, Republic of Korea

^2^Biomedical Implant Convergence Research Center, Advanced Institutes of Convergence Technology, Suwon 16229, Republic of Korea

^3^Department of Carbon and Nano Materials Engineering, Jeonju University, Jeonju 55069, Republic of Korea

*Corresponding Author

Cheol-Min Han, Jeonju University

Email : hancm@jj.ac.kr

Tel : +82-63-220-2208

Postal Address : 303 Cheonjam-ro, Wansan-gu, Jeonju-si, Jeollabuk-do, 55069, Republic of Korea


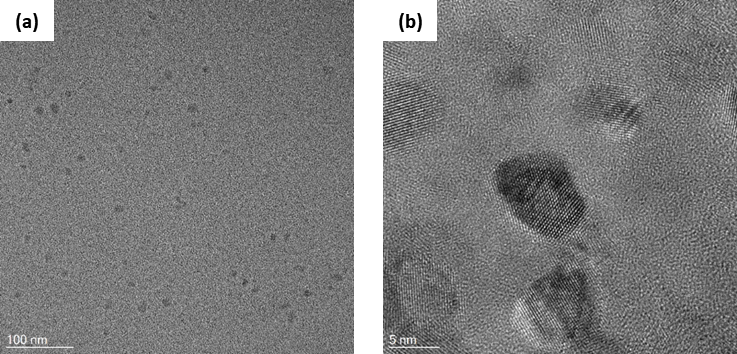


Figure S1. Transmission electron microscopy images of dried HA sol after 1 day of aging observed with (a) low (× 40,000) and (b) high (× 600,000) magnifications. Scale bars are (a) 100 nm and (b) 5 nm.


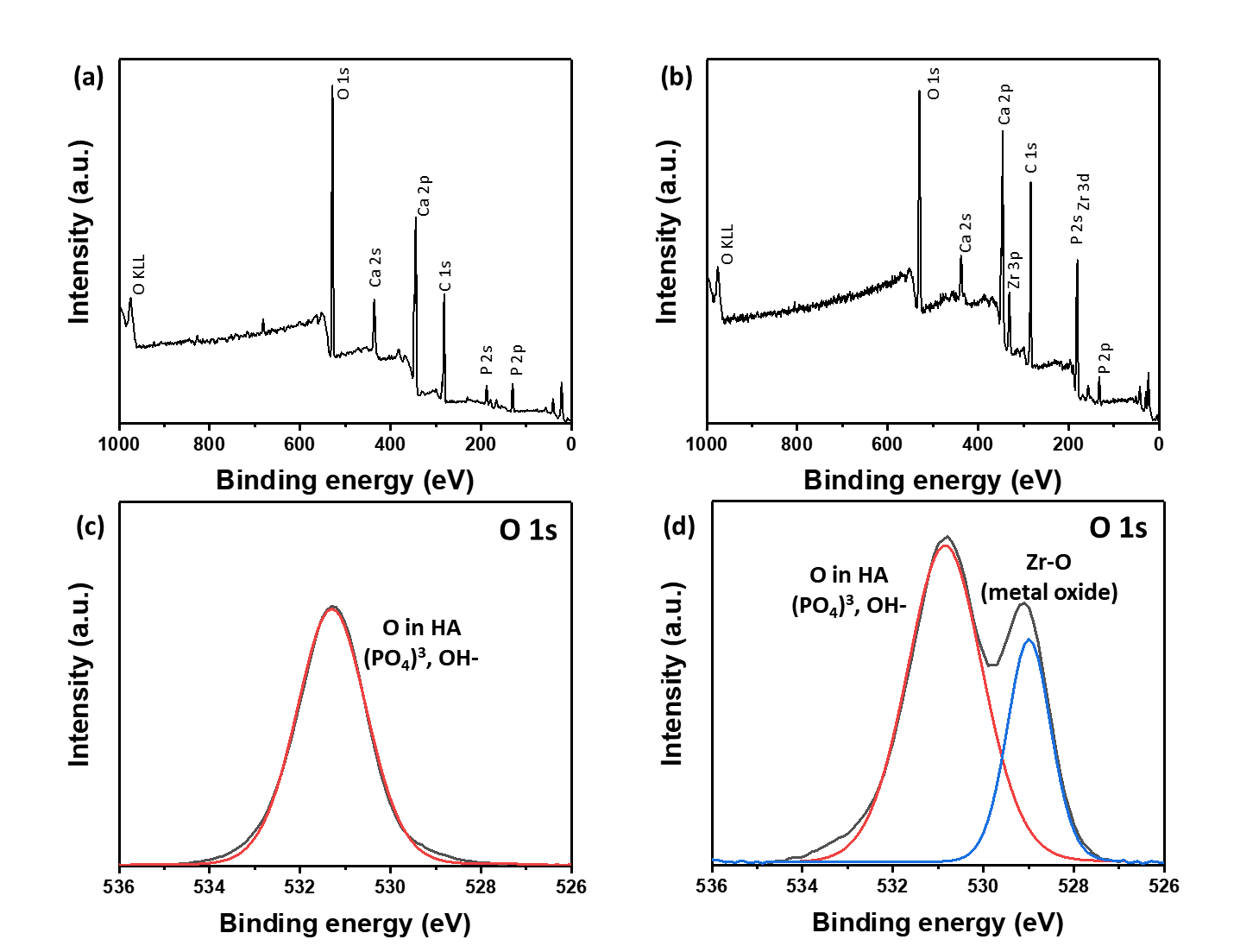


Figure S2. X-ray photoelectron spectroscopy data of (a, c) HA800 and (b, d) HA1000. (a, b : survey spectra, c, d : O 1s).


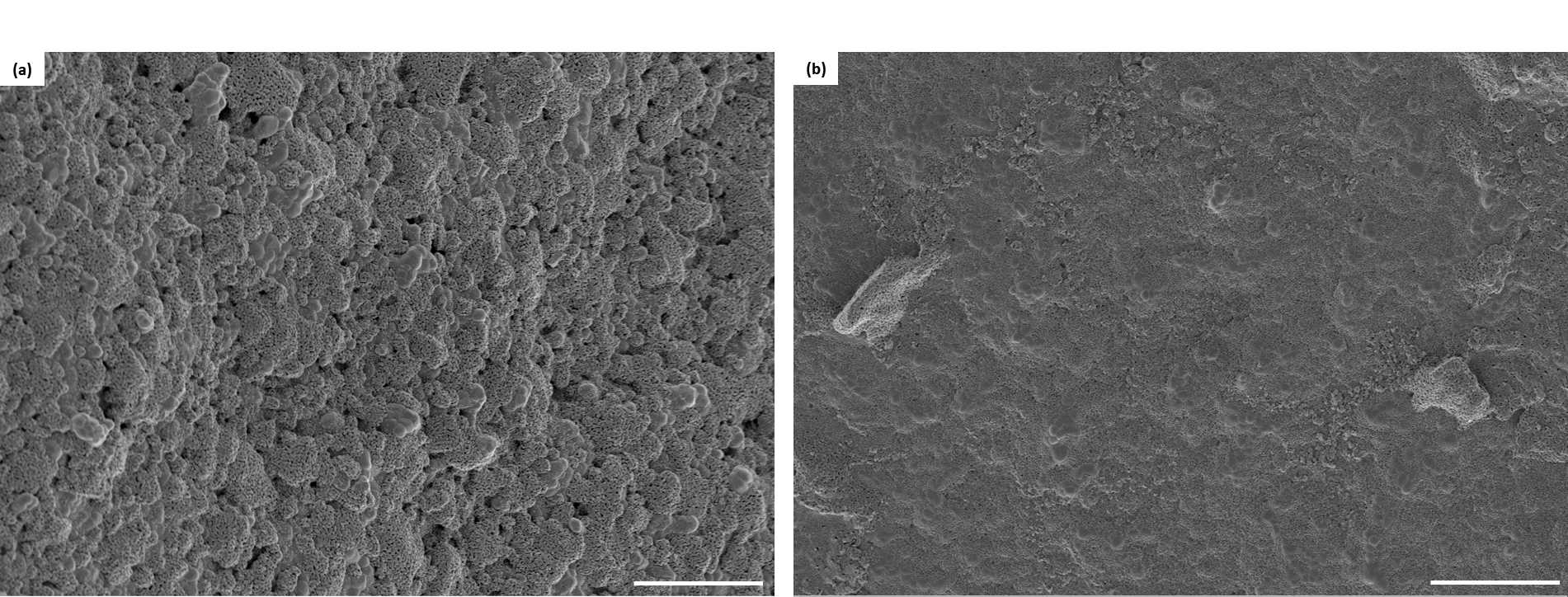


Figure S3. Scanning electron microscopy images of HA-coated zirconia screw sintered at 800 °C after implantation on synthetic bone; (a) ridge and (b) groove of screw. scale bars are 2 μm.
